# Supplementary material for: A Prospective Study of Etiology and Auditory Profiles in Infants with Congenital Unilateral Sensorineural Hearing Loss
Source: J Clin Med. 2022 Jul 7;11(14):3966. doi: 10.3390/jcm11143966 (PMC9324637; doi:10.3390/jcm11143966)
Supplement: Supplementary file 1 [file jcm-11-03966-s001.zip › jcm-1779420-supplementary.pdf]

Table S1, Supplementary Materials. Auditory Brainstem Response (ABR) mean and median latency difference by stimulus level with statistical significance. Wilcoxon's matched pairs test was used for paired comparisons and Mann-Whitney U test for the whole sample's interaural difference comparisons.

**Stimulus level (dB nHL)**			**40**	**50**	**60**	**70**	**80**
**Latency Wave V**	Interaural difference all	Mean (ms)	0.22	0.10	0.20	0.17	0.08
		Median (ms)	0.23	0.05	0.10	0.25	0.03
		p-value	0.38	0.64	0.45	0.12	0.59
		N (NE, IE)	16, 6	13, 10	9, 9	20, 12	9, 11
	Paired interaural difference	Mean (ms)	0.10	0.12	0.11	0.22	0.12
		Median (ms)	0.10	0.30	0.15	**0.25**	**0.13**
		p-value	0.75	0.35	0.47	0.03 *	0.04 *
		N	6	7	4	12	5
**Latency Wave III**	Interaural difference all	Mean (ms)		0.14	0.23	0.16	0.21
		Median (ms)		0.10	0.17	0.18	0.20
		p-value		0.68	0.69	0.18	0.09
		N (NE, IE)		11, 7	9, 5	20,11	9, 10
	Paired interaural difference	Mean (ms)		0.31	0.19	0.16	0.11
		Median (ms)		0.27	0.24	0.20	0
		p-value		0.29	0.47	0.11	0.14
		N		3	4	11	5
**Latency Wave I**	Interaural difference all	Mean (nV)				0.10	0.07
		Median (nV)				0.05	0.01
		p-value				0.93	0.72
		N (NE, IE)				17, 8	8,7
	Paired interaural difference	Mean (nV)				0.02	0.05
		Median (nV)				0.06	0.07
		p-value				0.89	0.27
		N				7	5

* $p < 0.5$.

Table S2, Supplementary Materials. Auditory Brainstem Response (ABR) mean and median amplitude difference by stimulus level with statistical significance. Wilcoxon's matched pairs test was used for paired comparisons and Mann-Whitney U test for the whole sample's interaural difference comparisons.

**Stimulus level (dB nHL)**			**40**	**50**	**60**	**70**	**80**
**Amplitude Wave V**	Interaural difference all	Mean (nV)	76	62	79	102	89
		Median (nV)	**74**	**58**	**69**	**101**	**56**
		p-value	0.0005 ***	0.002 **	0.02 *	0.0004 ***	0.03 *
		N (NE, IE)	16, 6	13, 10	9, 9	20, 12	9,11
	Paired interaural difference	Mean (nV)	63	60	87	100	67
		Median (nV)	**55**	38	85	**101**	4
		p-value	**0.03 ***	0.09	0.14	0.004 **	0.08
		N	6	7	4	12	5
**Amplitude Wave III**	Interaural difference all	Mean (nV)		46	52	67	68
		Median (nV)		30	63	54	115
		p-value		0.057	0.053	0.054	0.09
		N (NE, IE)		11, 7	9, 5	20, 11	9, 10
	Paired interaural difference	Mean (nV)		10	45	99	131
		Median (nV)		10	39	**104**	132
		p-value		1	0.14	0.02 *	0.50
		N		3	4	11	5
**Amplitude Wave I**	Interaural difference all	Mean (nV)				26	58
		Median (nV)				31	62
		p-value				0.24	0.07
		N (NE, IE)				17, 8	8,7
	Paired interaural difference	Mean (nV)				28	16
		Median (nV)				19	16
		p-value				0.18	0.50
		N				7	5

IE = impaired ear; NE = normal-hearing ear. * $p < 0.5$; ** $p < 0.01$; *** $p < 0.001$.
